# Biofortified Wheat Increases Dietary Zinc Intake: A Randomised Controlled Efficacy Study of Zincol-2016 in Rural Pakistan

**DOI:** 10.3389/fnut.2021.809783

**Published:** 2022-01-18

**Authors:** Nicola M. Lowe, Mukhtiar Zaman, Muhammad Jaffar Khan, Anna K. M. Brazier, Babar Shahzad, Ubaid Ullah, Gul Khobana, Heather Ohly, Martin R. Broadley, Munir H. Zia, Harry J. McArdle, Edward J. M. Joy, Elizabeth H. Bailey, Scott D. Young, Jung Suh, Janet C. King, Jonathan Sinclair, Svetlana Tishkovskaya

**Affiliations:** ^1^Centre for Global Development, University of Central Lancashire, Preston, United Kingdom; ^2^Department of Pulmonology, Rehman Medical Institute, Peshawar, Pakistan; ^3^Institute of Basic Medical Sciences, Khyber Medical University, Peshawar, Pakistan; ^4^Abaseen Foundation, Peshawar, Pakistan; ^5^School of Biosciences, University of Nottingham, Loughborough, United Kingdom; ^6^Research and Development Department, Fauji Fertilizer Company Ltd., Rawalpindi, Pakistan; ^7^Department of Nutritional Sciences, University of Nottingham, Loughborough, United Kingdom; ^8^Faculty of Epidemiology and Population Health, London School of Hygiene and Tropical Medicine, London, United Kingdom; ^9^Children's Hospital Oakland Research Institute, Oakland, CA, United States; ^10^Lancashire Clinical Trials Unit, University of Central Lancashire, Preston, United Kingdom

**Keywords:** zinc, biofortified wheat, rural Pakistan, zinc status, micronutrient intake, fatty acid, wheat flour

## Abstract

A new variety of zinc biofortified wheat (Zincol-2016) was released in Pakistan in 2016. The primary aim of this study was to examine the effects of consuming Zincol-2016 wheat flour on biochemical and functional markers of zinc status in a population with widespread zinc deficiency. An individually-randomised, double-blind, placebo-controlled cross over design was used. Fifty households were recruited to participate in the study, with each household included at least one woman of reproductive age (16–49 years) who was neither pregnant nor breast feeding or currently taking nutritional supplements. All households were provided with control flour for an initial 2-week baseline period, followed by the intervention period where households were randomly allocated in a 1:1 ratio to receive biofortified flour (group A; *n* = 25) and control flour (group B; *n* = 25) for 8-weeks, then switched to the alternate flour for 8-weeks. The trial has been registered with the ISRCTN (https://www.isrctn.com), ID ISRCTN83678069. The primary outcome measure was plasma zinc concentration, and the secondary outcome measures were plasma selenium and copper concentrations, plasma copper:zinc ratio and fatty acid desaturase and elongase activity indices. Nutrient intake was assessed using 24-h dietary recall interviews. Mineral concentrations in plasma were measured using inductively coupled plasma mass spectrometry and free fatty acids and sphingolipids by mass spectrometry. Linear Mixed Model regression and General Linear Model with repeated measures were used to analyse the outcomes. Based on an average flour consumption of 224 g/day, Zincol-2016 flour provided an additional daily zinc intake of between 3.0 and 6.0 mg for white and whole grain flour, respectively. No serious adverse events were reported. This resulted in significant, increase in plasma zinc concentration after 4 weeks [mean difference 41.5 μg/L, 95% CI (6.9–76.1), *p* = 0.02]. This was not present after 8 weeks (*p* = 0.6). There were no consistent significant effects of the intervention on fatty acid desaturase and elongase activity indices. Regular consumption of Zincol-2016 flour increased the daily zinc intake of women of reproductive age by 30–60%, however this was not associated with a sustained improvement in indices of zinc status.

## Introduction

The Pakistan National Diet and Nutrition surveys have reported widespread micronutrient deficiencies amongst women and children for decades, with those living in rural regions most at risk (1). Although the most recent survey, undertaken in 2018, reported an improvement in zinc status among women of reproductive age (WRA), zinc deficiency still affects 22.1% of WRA, along with vitamin A deficiency (27.3%), iodine deficiency (17.5%) and iron deficiency (18.2%) (2). Current strategies to improve micronutrient status in Pakistan include the National Food Fortification Programme that was launched in 2016, which aims to fortify wheat flour with iron, folic acid, zinc, and vitamin B12, and edible oil/ghee with vitamins A and D. However, the mid-term evaluation of the programme highlighted several challenges to the potential success of the fortification strategy, particularly with respect to the fortification of wheat flour. Key issues include the lack of capacity for effective monitoring and quality control of the fortification process, lack of mandatory legislation for flour fortification and weak consumer demand. In addition, around 20–30% of households (HHs) were found to consume flour milled at the large roller mills which were eligible to participate in the fortification programme, with the majority of HHs consuming flour milled at small local mills, known as “*Chakki*,” which fall outside of the current fortification programme and would be impossible to monitor due to the large number, many situated in hard-to-reach rural locations. Conversely, the oil and ghee vitamin fortification programme has a greater potential for success and is supported by government legislation and effective enforcement by the Punjab Food Authority in Punjab Province where much of the national oil and ghee is produced (3).

Biofortification of staple crops with key micronutrients is an alternative strategy for reaching some of the more remote areas of the country, where food fortification coverage is not practical (4, 5). This involves the enhancement of the nutrient content of the crop through traditional selective breeding techniques, genetic modification and/or agronomic techniques including the application of micronutrient fertilisers (4, 6). Globally, several biofortified crop varieties have been released, including iron-rich pearl millet in India (7), zinc-rich rice in Bangladesh (8), zinc-rich wheat in India and Pakistan (9), and vitamin A-rich sweet potato and maize in Africa (10).

To evaluate the success of an intervention, it is necessary to have a reliable and specific biomarker or health outcome measure for the target micronutrient(s). For zinc, this is particularly challenging at the individual level due to the lack of sensitivity and specificity of the indices currently used (11, 12). Plasma zinc concentration (PZC) is frequently used to assess zinc status in populations, however its concentration is under tight homeostatic control and at an individual level, the response to small changes in dietary zinc intake, such as those expected from the consumption of biofortified staples, are subtle, particularly when the additional zinc is consumed with food rather than taken as a supplement (13, 14). In addition, interpretation of PZC is complicated by the presence of concurrent infection, fasted or non-fasted state, and time of day (13, 15). Novel biomarkers have been explored, including enzymes involved in essential fatty acid (EFA) metabolism. Zinc acts as a cofactor in fatty acid desaturase and elongase enzymes, and recent studies have suggested that linoleic acid desaturation and elongation pathways may be sensitive to small changes in zinc intake (16–18). However, more studies are needed to explore this as a robust and sensitive functional indicator for zinc status, particularly from studies conducted in free-living, community settings where confounding co-morbidities may be present.

In Pakistan, a new variety of zinc biofortified wheat (Zincol-2016) was released by HarvestPlus in 2016. The BiZiFED programme was launched in 2017, with the overarching aim of exploring the potential for Zincol-2016 to improve dietary zinc intake with scale-up on a national level (19). The foundation phase of this programme included a double-blind, randomised controlled trial (RCT) with cross-over design. The primary aim of this RCT was to measure outcomes of consuming flour made from a zinc biofortified wheat grain variety, Zincol-2016, on dietary zinc intake and biomarkers of zinc status in a low-resource rural community setting in Pakistan. The secondary aim is to evaluate the potential usefulness of proposed novel biomarkers of zinc nutriture (20). In this paper we present the outcome of consuming Zincol-2016 on dietary zinc intake and plasma zinc and mineral concentrations in the study cohort. We also report the effect of the intervention on proposed novel functional zinc indicators, FADS1, FADS2, and ELOVL5, to explore their potential for the evaluation of the future, larger scale biofortification effectiveness trials.

## Materials and Methods

A double-blind, individually-randomised, placebo-controlled study with cross-over design was undertaken in a rural community in Pakistan between October 2017 and February 2018. The trial was registered with the ISRCTN registry, study ID ISRCTN83678069 and the study protocol has been published (20). Ethical approval was granted by the lead University (reference no. STEMH 697 FR) and the collaborating institution in Pakistan, Khyber Medical University. The study is reported according to CONSORT statement extension to randomised crossover trials (21).

The recruitment and consent process has been described previously (20). In brief, the target community was comprised of ~5,000 HHs, served by a Health Centre located near to the brick kilns close to Peshawar in Khyber Pakhtunkhwa (KP) province. The catchment area for this study was comprised of 10 villages, 5 of which were randomly selected for participation in the study. Ten HHs from each village were randomly selected and visited by the study manager to assess their eligibility and willingness to participate in the trial. The inclusion criteria were that the HH included a woman aged 16–49 years who was neither pregnant nor breastfeeding and not currently consuming nutritional supplements. There were no additional inclusion or exclusion criteria. If the head of the HH declined, another house from the same village was randomly selected and the invitation process repeated until 5 HHs had agreed to participate in each village. The primary outcome measure was PZC, and the target was to recruit 50 HHs, based on 5% significance level (two-sided) and 90% power, to detect an increase of plasma zinc concentration of 3.1 μg/dL with standard deviation (SD) 5.9 μg/dL taken from Hambidge et al. (22), with an attrition rate of 20%.

At the start of the 18-week protocol, HHs were randomised by a team member (MJK) to the intervention or control arm of the study using a block design whereby villages were blocks. One member of the team (MZam) who oversaw the logistics of the flour distribution but was not involved in the data collection or analysis, performed the allocation to the study arms. Flour distribution was undertaken by the store manager and community liaison officer, who also had knowledge of the allocation. The remaining team members and all participants were blinded to the allocation until data collection and preliminary statistical analyses were complete. The study comprised a 2-week baseline period, where all fifty HHs were provided with sufficient flour, milled from a standard wheat grain variety (Galaxy- 2013) to meet the HH needs. This was followed by two 8-week intervention periods, where HHs in the intervention arm (group A) received biofortified flour, milled from a zinc-rich variety of wheat (Zincol-2016) and those in the control arm (group B) continued to receive the standard wheat flour (Period 1). After 8 weeks the two groups crossed over with group A receiving the standard flour, and group B receiving the biofortified flour. A washout period between the two intervention periods was not required because part of the experimental design was to examine the shorter-term biomarker response to changes in dietary zinc intakes and zinc homeostatic mechanisms are known to respond rapidly to changes in dietary intake, so a “carry-over” effect was not expected. The participants were visited by the field team for data and sample collection at five timepoints (TP) during the study; during the baseline period (T1); the mid and endpoint of period 1 (T2 and T3, respectively); the mid and endpoint of period 2 (T4 and T5, respectively).

During the study, HHs were asked not to consume any other flour except that which was provided by the study team. Freshly milled flour was delivered to the HHs every 2 weeks, with sufficient quantity for all HH members based on self-reported HH consumption. Compliance was monitored by a member of the study field who visited each HH every 2 weeks to confirm the quantity of flour remaining. In addition, at the end of each timepoint, participants were asked for the number of occasions (meals) when she did not use the flour provided.

### Grain Production and Analysis

The grain used to produce both the standard (control) and biofortified flours (intervention) were grown under carefully controlled conditions at a farm in Punjab province. Two genotypes of grain, a standard variety (Galaxy-2013) and a biofortified variety (Zincol-2016) were sown in November 2016 and harvested in May 2017 by our project partner, Fauji Fertilizer Company Ltd. Zincol-2016 had been selectively bred by HarvestPlus for its zinc accumulation properties as well as its resilience to common pests and pathogens, mainly fungal diseases. To increase the potential for zinc uptake into the grain, which is dependent on soil zinc availability (9), zinc fertiliser (Zn 13% as EDTA Zn) was applied to the soil (1.25 kg/ha) before sowing the crop; and foliage (ZnSO_4_·H_2_O 33% Zn, applied at 1 kg/ha of product dissolved in 250 L of water) four times during the booting and heading stage. The Galaxy-2013 wheat was grown under standard conditions without additional zinc. Both varieties of grain were manually harvested, threshed mechanically on site and the grain collected into pre-labelled sacks which were transported by road to the study field site in KP. Each sack of grain was manually cleaned to remove any straw and grit before being sent for milling at a local commercial flour mill. A sample of grain (10 g) from the bottom, middle and top of each sack was collected for analysis of the mineral content. An aliquot of each sample was transported to the UK for mineral analysis at the University of Nottingham (UoN) using methods previously described (9, 23). In brief, whole grain was pre-soaked overnight at room temperature in 70% Trace Analysis Grade (TAG) HNO_3_ and 2 mL H_2_O_2_. Samples were then microwave digested (Multiwave 3000 microwave system, Anton Paar Gmbh, Graz, Austria) and the whole-grain zinc and other mineral concentrations determined by inductively coupled plasma-mass spectrometry (ICP-MS; Thermo Fisher Scientific iCAPQ, Thermo Fisher Scientific, Bremen, Germany).

### Participant Characteristics and Haematology

The characteristics of the participants, including indicators of socioeconomic status, HH demographics and anthropometric measures of the participating WRA were collected at baseline. These have been reported previously along with dietary diversity data (24). In addition, blood samples were collected at each T to monitor health status throughout the study. Whole blood (non-fasting) was drawn from the antecubital vein through a butterfly needle into plastic vacutainers (BD Diagnostics, Switzerland). Blood (2 mL) was collected into a tube containing Ethylenediaminetetraacetic acid (EDTA) anticoagulant for red blood cell count (RBC), haematocrit, haemoglobin (Hb), mean corpuscular volume (MCV) and mean corpuscular haemoglobin concentration (MCHC). These were measured on whole blood using an automated haematology analyzer (Sysmex XP-100, 19 Jalan Tukang, Singapore).

### Dietary Analysis

Diet was assessed using five 24-h recalls collected during the 18-week protocol, at all five timepoints. A minimum of two 24-h recalls are required to estimate nutrient intakes (25). The intention was to establish the usual nutrient intakes in the community, not to examine the effect of the intervention on diet, thus the mean of the five timepoints were used to evaluate intakes in comparison with the dietary guidelines for Pakistan and also to identify any statistically significant differences in nutrient intakes between the two groups that may be confounding factors in the interpretation of the outcome measures. The dietary recalls were conducted by the study nutritionist (GK) using the multiple pass method and portion sizes were estimated using HH measures. In addition, detailed recipes for composite meals were collected to enable accurate entry of ingredients into the nutrient database (Windiets 2017). The Windiet database was augmented using food composition data from Bangladesh (26), and Pakistan (27) to improve the accuracy of the nutrient composition data for foods grown in the region. In addition, white beans, kidney beans and lentils, which are commonly consumed zinc containing foods in the study location, were purchased from the local market and the zinc content measured by ICP-MS at UoN. These values were added to the Windiet database and used in the dietary analysis. Values for the phytate content of individual food items were input manually from the Indian food composition database (28).

### Plasma Mineral and Essential Fatty Acid Analyses

For plasma trace mineral analysis, non-fasting whole blood (5 mL) was collected at all five timepoints into trace-element-free tubes containing EDTA anticoagulant. Blood plasma was separated by centrifugation within 40 min of sample collection and stored at −80°C at Khyber Medical University prior to shipping on dry ice to UoN and Children's Hospital Oakland Research Institute (CHORI). Elemental concentrations of zinc in plasma samples were determined at UoN using inductively coupled plasma–mass spectrometry (ICP-MS; Thermo Fisher Scientific iCAPQ, Thermo Fisher Scientific, Bremen, Germany). Full details of the instrument conditions and quality control have been published previously (24). Essential fatty acid (EFA) concentrations in the plasma were measured at CHORI. An Infinity Quaternary liquid chromatography system in tandem with a 6490 Triple Quadrupole mass spectrometer (Agilent Technologies) was used to simultaneously quantify total plasma 18:2n−6 linoleic acid (LA), 18:3n−6 γ -linolenic acid (GLA), 20:3n−6 dihomo-γ -linolenic acid (DGLA), and 20:4n−6 arachidonic acid (ARA) as previously described (14). The proxy indices of the activity of enzymes involved in EFA metabolismwere determined from the ARA:DGLA (FADS1), GLA:LA (FADS2) and DGLA:LA (ELOVL5) ratios, respectively.

### Statistical Analysis

The analyses were done on intention to treat basis, as a complete case analysis. Data were analysed using IBM SPSS Statistics Version 28 (Armonk, NY: IBM Corp). The statisticians were blinded to the intervention assignment until the analysis of the primary outcome was complete.

### Primary Outcome Analysis

To test for an intervention effect, unadjusted analysis was performed with a paired samples *t*-test based on within participant differences for a PZC in two study periods. The normality assumption was checked and according to Kolmogorov-Smirnov and Shapiro-Wilk tests PZC data were normally distributed.

To explore the effect of the intervention on the primary outcome adjusted for baseline and over all timepoints, Linear Mixed Model (LMM) regression was used which provides a general and flexible approach to accommodate both fixed and random effects. The models included the treatment group (Intervention vs. Control), time and the treatment period (sequence) as fixed effects and the participant as a random effect. PZC at baseline was included as a continuous covariate. The model was chosen on the basis of Bayesian information criteria. To undertake the preliminary tests for carryover effect and period effect, required in cross-over trials (29, 30), a sequence parameter and period parameter were added into the model. Analysis for treatment effect at end points, T3 and T5, was complemented with comparisons of within-subject differences at interim collection points, T2 and T4, and across all four timepoints (T2–T5). An interaction term between study period and intervention effect was considered for inclusion in the model but was not found to be statistically significant so was therefore removed from the final model.

### Secondary Outcome Analyses

The intervention effect for the secondary outcomes (plasma mineral concentrations of selenium, copper and copper:zinc ratio) was tested using a LMM with the same fixed and random effects as for the primary outcome, adjusted for baseline. General Linear Model (GLM) analyses with repeated measures were used to explore the within participant effects of time on consuming biofortified flour. According to the most recent dietary recommendations for zinc which were published by the European Food Safety Authority (EFSA) (31), based on a diet of >1,200 mg phytate per day, the Reference Nutrient Intakes (RNI) for WRA is 12.7 mg zinc per day. The Pakistan Dietary Guideline for Better Nutrition (32) set the recommended daily allowances for WRA at 20 mg zinc per day. Therefore, one-sample *t*-tests were utilised to compare the participants' dietary intake of zinc against the reference standards for both the EFSA and Pakistan Dietary Guideline for Better Nutrition. Overall mean nutrient intake between the two study groups were compared using an independent sample *t*-test.

Finally, to explore the linear association between PZC and FADS1 at T3 and T5, Pearson's product moment correlation analyses were adopted.

## Results

**Figure 1:**
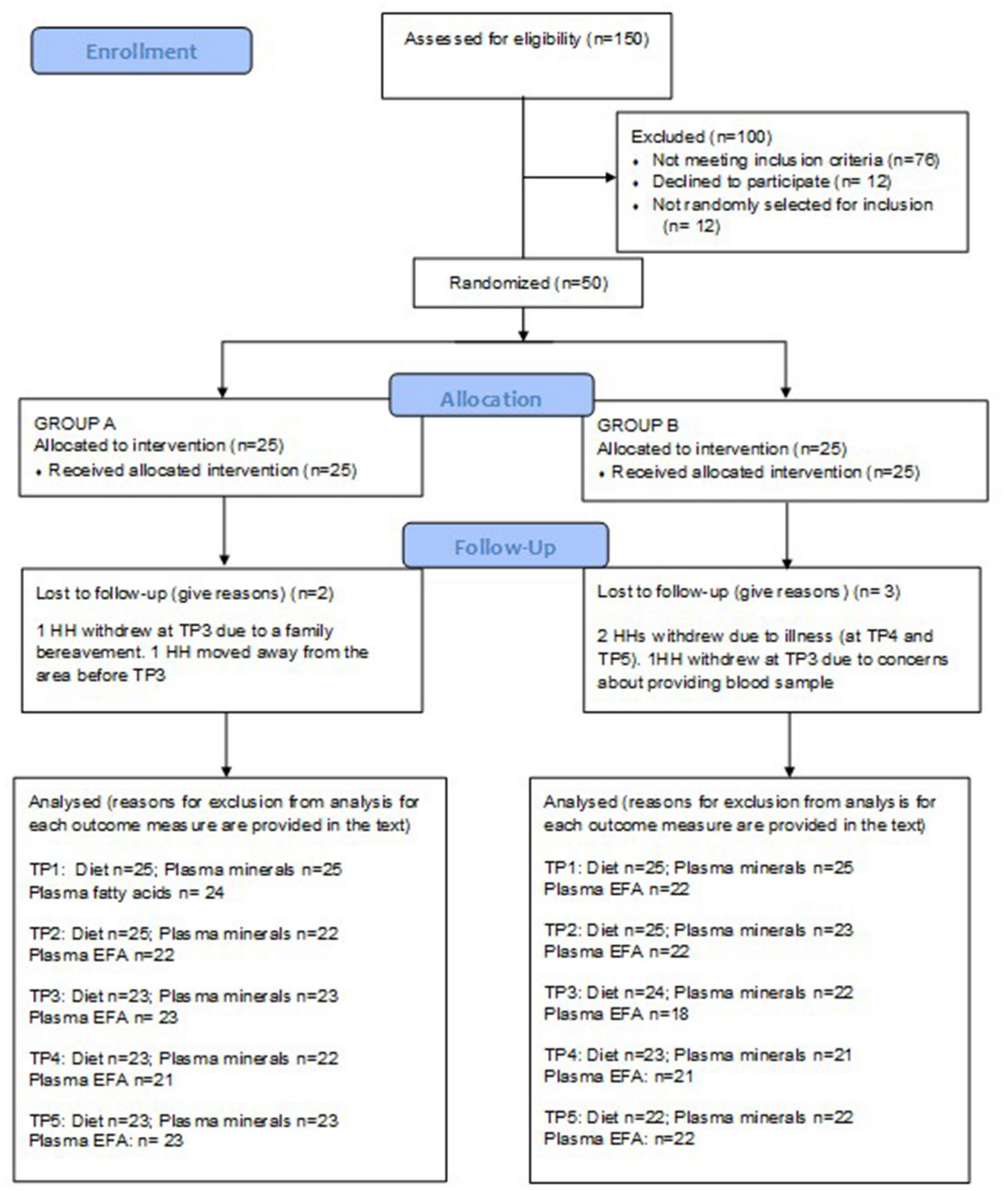
CONSORT flow diagram.

One hundred and fifty households were assessed for eligibility. Fifty households were recruited to participate in the study which began in October 2017 and ended in February 2018. Baseline participant characteristics have been reported elsewhere (24). Five participants withdrew from the study. The reasons for attrition were: unwillingness to provide a blood sample (*n* = 2), migration out of the area (*n* = 1), and severe illness (*n* = 2). The CONSORT flow diagram is provided in Figure 1. Some blood samples were lost to individual outcome measure analyses due to non-viability of the sample for various reasons including haemolysis or low sample volume. The number of samples analysed for each outcome are provided in Figure 1. The HH adherence to the protocol was good overall. There were no occasions reported during T1 or T3 where study flour was not used for every meal. At T2, one HH reported use of non-study flour for three meals and one HH for 2 meals. At T4, HHs reported using non-study flour for two (1 HH) or three meals (3 HHs), and at T5, there were reports of the use of non-study flour for two (2 HHs) or three (1 HH) meals. In this timepoint, one HH reported not using the study flour for 30 meals (10 days). Incidences of non-compliance were distributed between both study groups, with one from each group at T2, two from each group at T4 and three from group A and two from group B at T5. The HH with the long period of non-compliance was in the control period when this occurred. The HHs reporting non-compliance were different at each of the timepoints.

### Diet Analysis

The average macronutrient and zinc intakes for Groups A and B are presented in Table 1. The zinc intakes are based on the database analyses, which do not take into consideration the additional zinc intake from the biofortified flour. There were no significant differences in the overall mean (T1–T5) nutrient intakes between the two groups. No participants reported supplement use during the RCT.

**Table 1:** Daily dietary nutrient and phytate intake during the randomised controlled trial.

	**Timepoint 1** ^**^¶^**^		**Timepoint 2** ^**^¶^**^		**Timepoint 3** ^**^¶^**^		**Timepoint 4** ^**^¶^**^		**Timepoint 5** ^**^¶^**^		**All timepoints, 1–5**	
	**Mean**	**SD**	**Mean**	**SD**	**Mean**	**SD**	**Mean**	**SD**	**Mean**	**SD**	**Mean**	**SD**
**Intervention group A^§^**	**(*****N*** **=** **25)**		**(*****N*** **=** **25)**		**(*****N*** **=** **23)**		**(*****N*** **=** **23)**		**(*****N*** **=** **23)**			
Energy (kcal/d)	2,002	721	2,071	484	2,076	721	2,081	568	2,079	510	2,048	424
Fat (g/d)	73.7	35.6	69.6	17.7	74.9	37.6	74.8	34.7	74.5	25.3	73.4	17.5
Protein (g/d)	60.3	30.2	60.3	27.6	60.5	32.5	61.1	29.6	64.1	35	61.1	19.3
Carbohydrate (g/d)	266.1	103.7	293	78	281.2	104.9	282.7	82.4	279.9	90.9	277.6	65.2
Iron (mg/d)	17.3	9.2	18.2	7.2	18.7	8.2	18.7	8.2	19.2	9.1	18	5.7
Zinc (mg/d)	9.6	5.5	9.8	4.2	9.8	4.9	9.7	4.6	10.4	5.1	9.7	3.3
Phytate (mg/d)	1,455	804	1,711	779	1,489	833	1,530	786	1,335	694	1,482	562
Phytate/Zn (molar ratio)	15.3	3.4	17.2	3.9	15.2	5	15.7	3.3	12.9	3	15.3	1.9
**Intervention group B** ^**^§^**^	**(*****N*** **=** **25)**		**(*****N*** **=** **25)**		**(*****N*** **=** **24)**		**(*****N*** **=** **23)**		**(*****N*** **=** **22)**			
Energy (kcal/d)	2,077	821	2,130	520	2,087	714	2,169	603	2,106	671	2,101	459
Fat (g/d)	83.3	41.6	65.7	28.7	71.5	25.7	68.9	22	77.9	33.2	73.2	15.8
Protein (g/d)	64.8	37.9	69.6	23.9	66.1	40.2	70.8	30.2	59.5	25.6	65.4	17.7
Carbohydrate (g/d)	259.9	118	307.4	67.1	286.9	127.9	308.1	101.9	284	100.1	287.4	68.3
Iron (mg/d)	19.3	12.3	20.3	7.7	19.3	10.8	21.6	8.7	17	6.2	19.4	5.2
Zinc (mg/d)	10.4	5.7	11.2	4.3	10.4	6.1	11.1	4.3	9.1	3.6	10.4	2.6
Phytate (mg/d)	1,295	664	1,741	642	1,562	1,065	1,471	564	1,311	550	1,469	411
Phytate/Zn (molar ratio)	13.2	4.2	16	4.6	14.4	5	13.6	3.9	14.5	3.2	14.5	2.1

§ *Intervention group A received biofortified flour in period 1 and control flour in period 2 of the study. Intervention group B received control flour in period 1 and biofortified flour in period 2 of the study*.

¶ *Timepoint 1 = baseline; Timepoint 2 = week 4 of period 1; Timepoint 3 = week 8 of period 1; Timepoint 4 = week 4 of period 2; Timepoint 5 = week 8 of period 2*.

Average nutrient intakes for all participants (Groups A and B combined) were compared with dietary recommendations (Table 2). The mean ± SD daily energy intake was 2,074 ± 483 kcal which is commensurate with recommended intakes women with low to moderate activity levels of 1,816–2,234 kcal according to the Pakistan dietary guidelines (32).

**Table 2:** Nutrient intakes for all participants compared with daily dietary recommendations.

	**Nutrient intake**		**% Energy**		
**All timepoints**	**Mean**	**SD**	**Mean**	**SD**	**PK guidelines**
Energy (kcal/d)	2,074.3	438.3			2,160 kcal/d
Fat (g/d)	73.3	16.5	28.7	4.3	
Protein (g/d)	63.2	18.4	12.1	2.9	0.52/kg = 34.4 g^*^
Carbohydrate (g/d)	282.5	66.3	54.3	4.0	
Iron (mg/d)	18.7	5.4			30 mg/d
Zinc (mg/d)	10.1	3.0			20 mg/d
Phytate (g/d)	1,475.5	487.3			
Phytate/Zn (molar ratio)	14.9	2.0			

* *Mean weight at baseline = 66.3 kg*.

The overall mean ± SD dietary intake of zinc was 10.1 ± 3.0 mg per day, and phytate intake averaged 1,476 ± 487 mg per day, giving a mean phytate to zinc molar ratio of 14.9. One sample *t*-tests showed that dietary zinc intake was significantly lower than the EFSA recommendations of 12.7 mg/d (*p* < 0.001) and Pakistan Dietary Guideline for Better Nutrition value of 20 mg/d (*p* < 0.001). Based on the mean values for all timepoints completed, only 18% of the participants (9 of the 50) met the ESFA recommended intake, and none met the Pakistan guidelines for either zinc (or iron). For participants in this study, bread contributed almost 40% to the daily energy intake.

### Grain Analysis

A total of 203 samples of Galaxy and 172 samples of Zincol-2016 grain were analysed for zinc content. Exploratory statistical analysis of the data revealed that for each wheat variety, a number of outliers were identified which we suspect were due to mislabeling of some of the sample bags during the aliquoting stage for transport to the UoN (UK). Outliers were therefore removed from the analysis if the zinc content was >1.5 × the interquartile range for the group. Based on this, 32 samples of Galaxy and 33 samples of Zincol-2016 were removed from the final data analysis, reported in Table 3.

**Table 3:** Zinc content of control (Galaxy) and biofortified (Zincol-2016) wheat grain.

**Wheat variety**	***N***	**Mean (Zinc) mg/kg**	**SD**	**Min**	**Median**	**Max**	**95% CI**
Zincol-2016	139	49.3	5.6	27.3	49.7	61.3	48.3–50.2
Galaxy	171	22.2	2.9	14.3	22.2	30.9	21.7–22.6

### Estimation of the Daily Zinc Intake From Whole Wheat Flour

The average bread consumption of the women participating in this trial was 324 g per day, taken from the 24-h recalls. Using a local recipe, naan bread contains 69 g of dry wheat flour per 100 g fresh-weight bread, giving an estimated average wheat flour consumption per day of 224 g. Thus, if whole grain Zincol-2016 is consumed, then the daily intake of zinc from flour is 224 ×0.0493 = 11.0 mg. If whole grain Galaxy is consumed, then the daily intake of zinc from flour is 224 ×0.0222 = 5.0 mg (rounded to 1 decimal place). On the basis of consuming whole grain flour, the additional dietary zinc provided by Zincol-2016 is 6.0 mg per day (rounded to 1 decimal place).

The zinc content of the flour used for baking depends on the amount of bran retained in the flour during the milling process, and the treatment of the flour at the HH level. For this study, the flour was provided “whole” without the bran removed. However, at the HH level, it is common practise for the women to sieve the flour to remove some, or all, of the bran depending on the coarseness of the sieve used and the desired outcome. For example, a fine sieve may be used to produce the whitest flour when baking Paratha, but a coarse sieve used for flour used in the baking of naan or roti. Since the zinc concentration of the bran is typically 3 times greater than that of the white flour (23) and the bran constitutes approximately 25% of the grain weight, removing the bran will reduce the overall zinc content of the flour by ~50%. Therefore, with all the bran removed, the contribution to daily zinc intake from white flour is estimated to be 5.5 mg for Zincol-2016 and 2.5 mg for Galaxy, a difference of 3.0 mg per day. The increase in daily zinc intake from consuming 224 g biofortified flour per day is estimated to be within the range 3.0 to 6.0 mg per day depending on the bran content of the flour.

### Plasma Mineral Analysis

Mean plasma zinc, copper, and selenium concentrations measured at each of the 5 timepoints are presented in Table 4.

**Table 4:** Plasma Zinc, selenium, and copper concentrations (μg/L) and copper:zinc ratio for groups A and B at each timepoint.

**^**^¶^**^T**	**Flour**		**Zinc (μg/L)**		**Selenium (μg/L)**		**Copper (μg/L)**		**Copper:Zinc**
		**N**	**Mean**	**SD**	**Mean**	**SD**	**Mean**	**SD**	**ratio**
**Group A**									
T1	Control	25	690.8	118.2	95.8	13.7	1,084.7	207.2	1.61
T2	Zn Biofortified	22	664.0	100.8	90.1	13.6	988.5	231.8	1.50
T3	Zn Biofortified	23	670.7	114.0	94.6	15.4	1,035.6	252.2	1.56
T4	Control	22	634.2	93.7	88.7	11.6	902.2	168.6	1.44
T5	Control	23	572.2	89.4	84.2	12.9	904.5	219.4	1.61
**Group B**									
T1	Control	25	702.3	119.7	97.1	19.6	1,066.0	226.4	1.57
T2	Control	23	621.2	103.7	95.1	16.4	1,027.8	357.2	1.68
T3	Control	22	685.0	107.1	106.4	14.5	1,016.3	209.7	1.52
T4	Zn Biofortified	21	685.9	131.1	98.2	13.4	1,010.4	259.7	1.49
T5	Zn Biofortified	22	601.5	73.4	88.0	15.2	915.2	212.0	1.54

¶ *T = Timepoint, where Timepoint 1 = baseline; Timepoint 2 = week 4 of period 1; Timepoint 3 = week 8 of period 1; Timepoint 4 = week 4 of period 2; Timepoint 5 = week 8 of period 2*.

#### Primary Outcome Measure PZC

**Figure 2:**
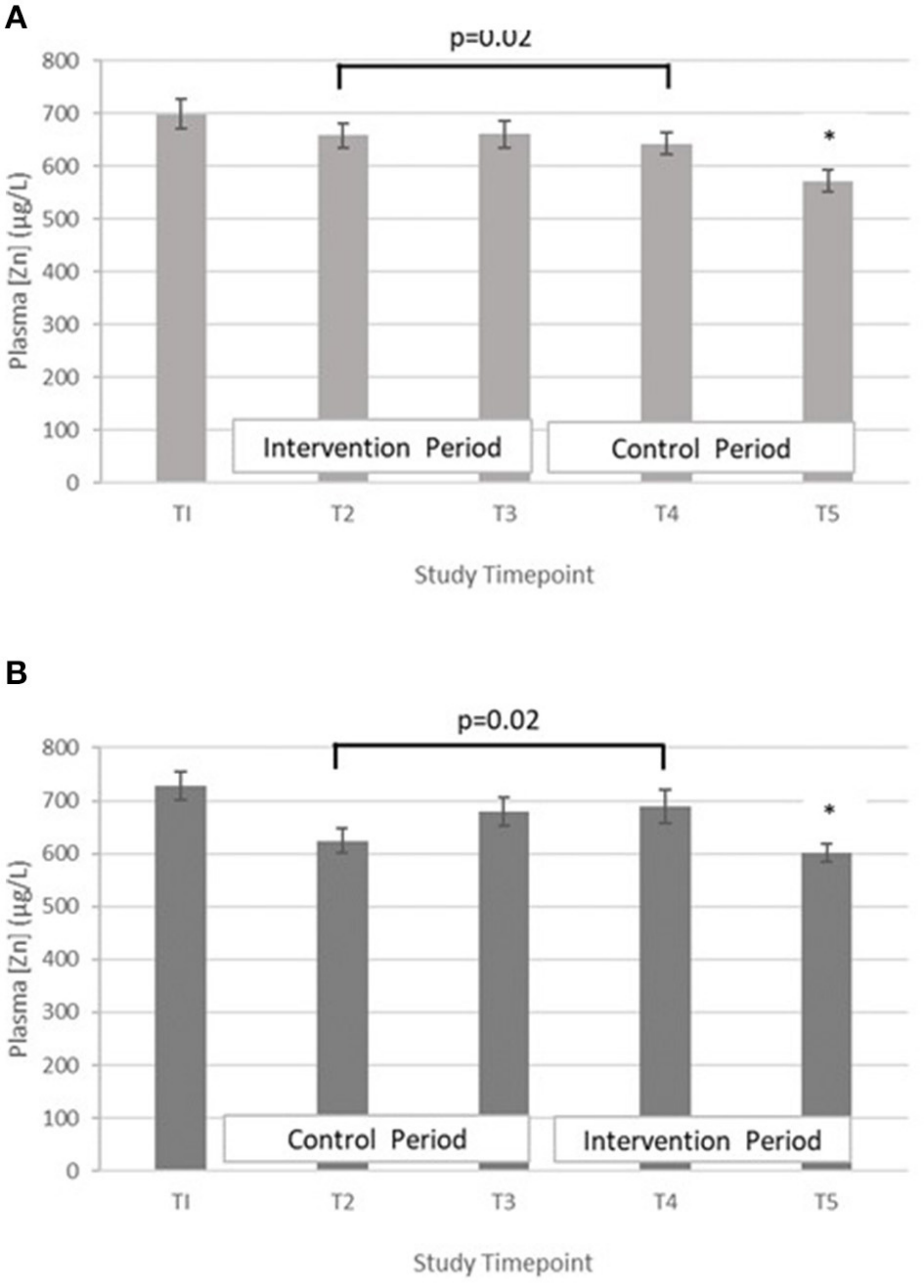
**(A)** Plasma zinc concentration measured in Group A at baseline (T1), week 4 of period 1 (T2), week 8 of period 1 (T3), week 4 of period 2 (T4) and week 8 of period 2 (T5). Bars indicate estimated marginal means with standard error. *Estimated marginal mean is significantly lower than at all other timepoints, *p* < 0.01. **(B)** Plasma zinc concentration measured in Group B at baseline (T1), week 4 of period 1 (T2), week 8 of period 1 (T3), week 4 of period 2 (T4) and week 8 of period 2 (T5). Bars indicate estimated marginal means with standard error. *Estimated marginal mean is significantly lower than at all other timepoints, *p* < 0.01.

Testing differences within participants at T3 and T5 for treatment effect with a paired t-test revealed that there was no evidence for a treatment effect [*t* = 0.77 (42 df) and *p* = 0.45]. When the analysis at mid points of period 1 and period 2 (T2 and T4) were performed, evidence of a treatment effect with statistically significant within-subject differences in PZC were demonstrated [*t* = 2.42 (40 df), *p* = 0.02]. The mean difference between the intervention and control at mid points T2 and T4 was 41.5 μg/L (SD = 109.7) and mean differences between T2 and T4 in Group A and B were 15.5 μg/L (SD = 118.1) and −66.2 μg/L (SD = 97.4), respectively (Figure 2).

From the LMM adjusted for PZC at baseline, the paired differences between the cross over intervention and control groups for the primary end points T3 and T5 are presented in Table 5.

**Table 5:** Primary end point (T3 and T5) analysis of differences between intervention and control groups.

**Plasma mineral**	**Intervention, mean (SD), *n* = 45**	**Control, mean (SD), *n* = 45**	**Paired differences^∧^, mean with 95% CI**	***p*-value**
Zinc (μg/L)	636.8 (101.5)	627.3 (113.8)	10.6 (−32.6, 53.8)	0.62
Selenium (μg/L)	91.4 (15.5)	95.0 (17.6)	−3.6 (−9.4, 2.2)	0.22
Copper (μg/L)	976.7 (238.7)	959.2 (219.7)	11.0 (−64.9, 86.9)	0.77
Copper:zinc ratio	1.6 (0.4)	1.6 (0.4)	−0.03 (−0.2, 0.09)	0.63

∧ *Intervention minus control. P-values are obtained using Linear Mixed Model adjusted for baseline*.

The observed *p*-value at the sequence parameter in the LMM (*p* = 0.46) indicates that the carry-over treatment effect is statistically insignificant, in agreement with the study assumption that the washout period is not required between the two periods.

To explore the trend of PZC over all four timepoints (T2–T5) (period effect), time was included in the LMM. There was a statistically significant difference in PZC between the four timepoints. Compared to T5, the PZC was greater at T2, T3, and T4, with mean increases of 53.8 μg/L (95% CI 17.6, 90.1 μg/L, *p* = 0.004), 88.2 μg/L (95% CI 52.0,124.5 μg/L, *p* < 0.001) and 73.6 μg/L (95% CI 37.0, 110.1 μg/L, *p* < 0.001), respectively.

#### Secondary Outcome Measures

The results of the LMM for plasma mineral outcomes are presented in Table 5.

**Figure 3:**
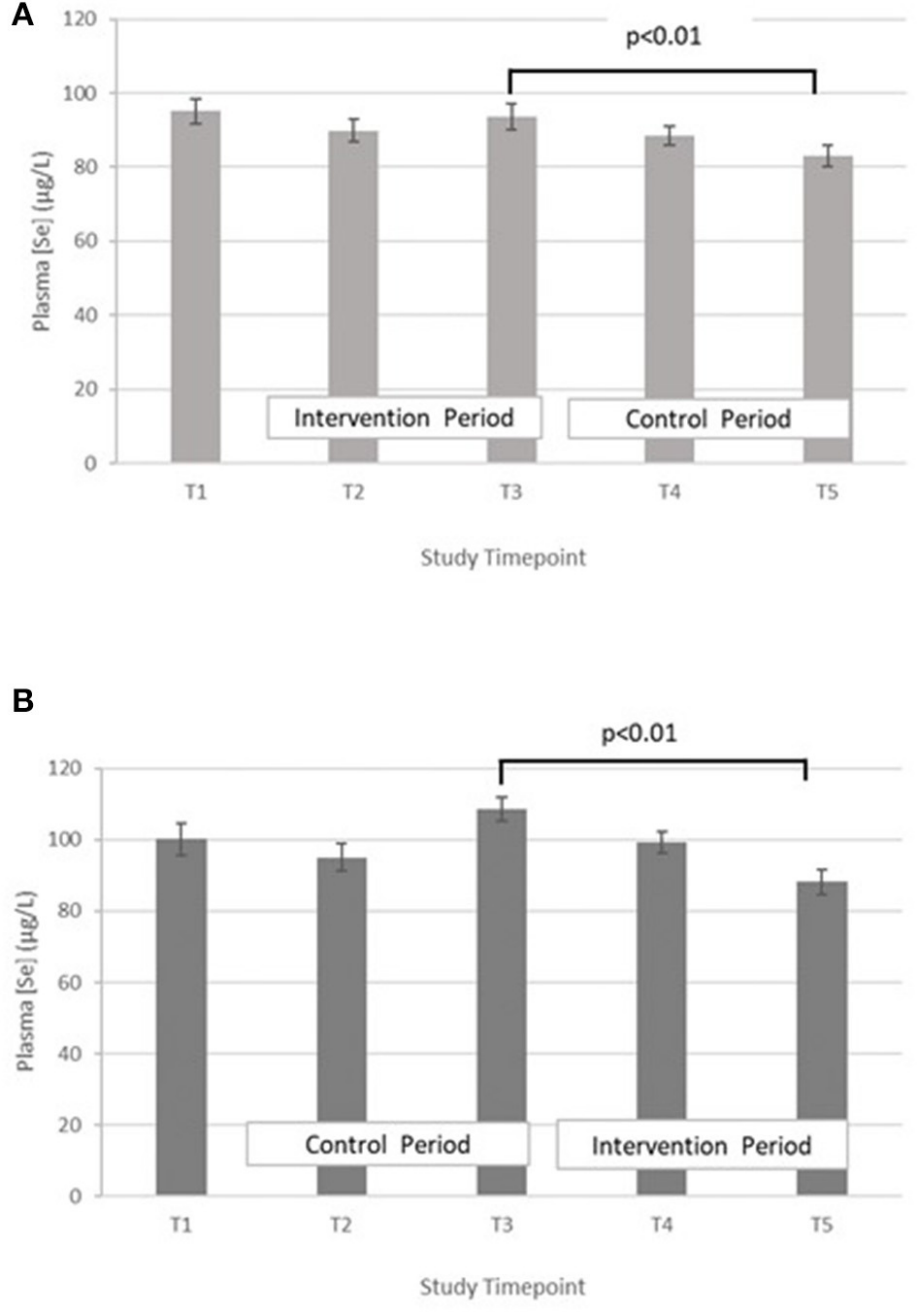
**(A)** Plasma selenium concentration measured in Group A at baseline (T1), week 4 of period 1 (T2), week 8 of period 1 (T3), week 4 of period 2 (T4) and week 8 of period 2 (T5). Bars indicate estimated marginal means with standard error. **(B)** Plasma selenium concentration measured in Group B at baseline (T1), week 4 of period 1 (T2), week 8 of period 1 (T3), week 4 of period 2 (T4) and week 8 of period 2 (T5). Bars indicate estimated marginal means with standard error.

Selenium – The treatment effect was not statistically significant. GLM with repeated measures indicated that in group A the plasma selenium concentration was significantly lower at T5 compared with T3 (*p* < 0.01). There were no differences between T2 and T4. A similar pattern was seen within group B, with Se at T5 being significantly lower than T3 (*p* < 0.01), but no significant differences between T2 and T4 (Figure 3).

**Figure 4:**
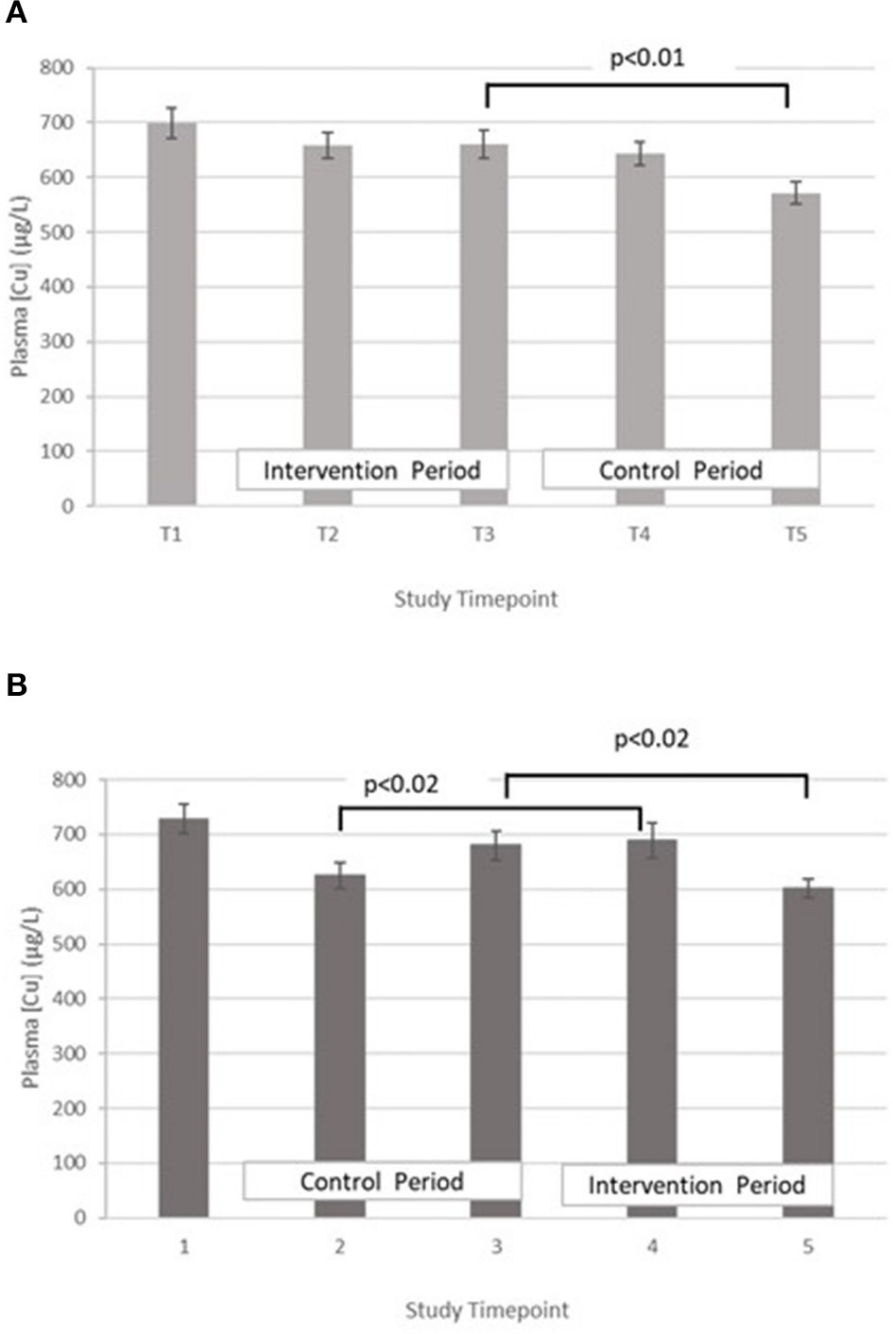
**(A)** Plasma copper concentration measured in Group A at baseline (T1), week 4 of period 1 (T2), week 8 of period 1 (T3), week 4 of period 2 (T4) and week 8 of period 2 (T5). Bars indicate estimated marginal means with standard error. **(B)** Plasma copper concentration measured in Group B at baseline (T1), week 4 of period 1 (T2), week 8 of period 1 (T3), week 4 of period 2 (T4) and week 8 of period 2 (T5). Bars indicate estimated marginal means with standard error.

Copper – The treatment effect was not statistically significant. GLM with repeated measures indicated that in group A plasma copper concentration was significantly higher at T3 vs. T5. Within group B, plasma copper concentration was significantly greater in T3 vs. T5 and T4 vs. T2 (Figure 4).

**Figure 5:**
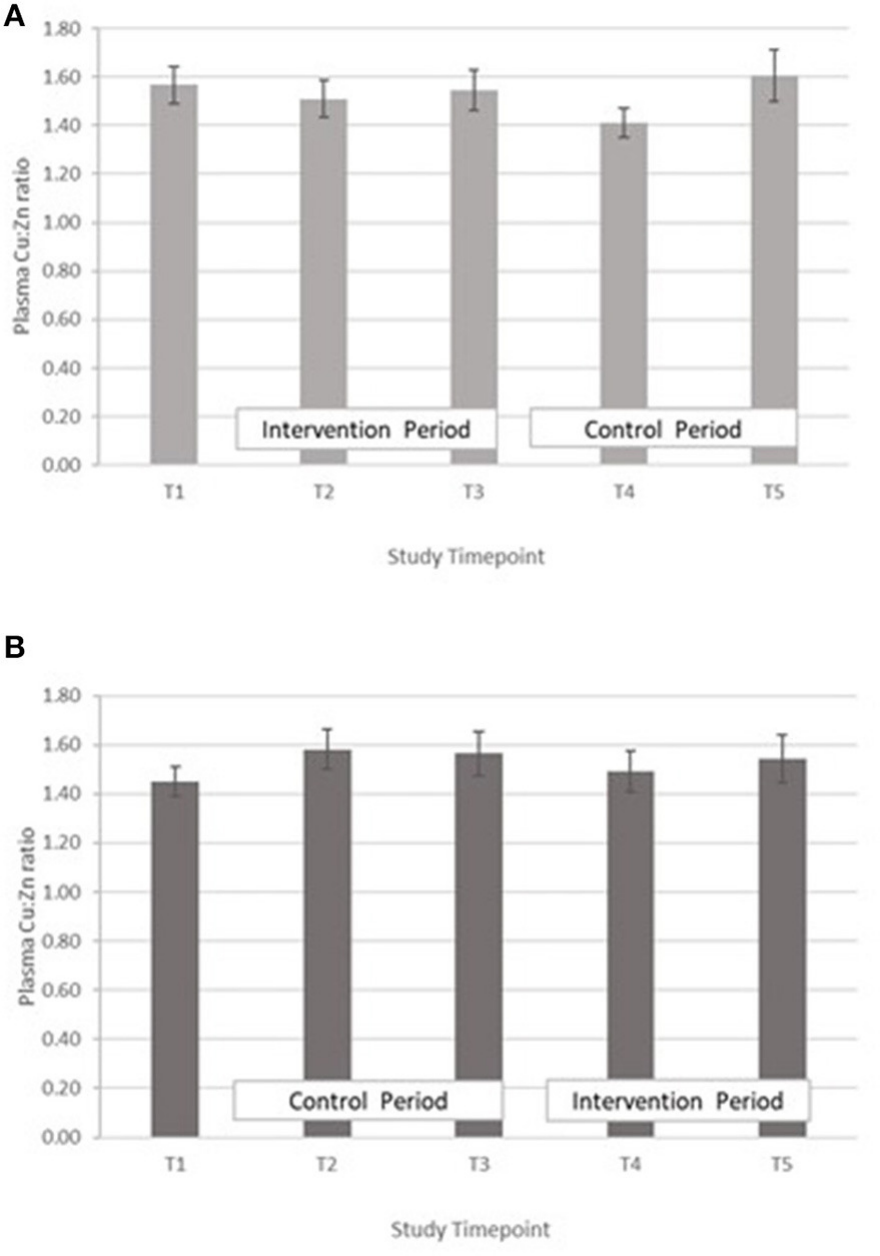
**(A)** Plasma copper:zinc ratio measured in Group A at baseline (T1), week 4 of period 1 (T2), week 8 of period 1 (T3), week 4 of period 2 (T4) and week 8 of period 2 (T5). Bars indicate estimated marginal means with standard error. **(B)** Plasma copper:zinc ratio measured in Group B at baseline (T1), week 4 of period 1 (T2), week 8 of period 1 (T3), week 4 of period 2 (T4) and week 8 of period 2 (T5). Bars indicate estimated marginal means with standard error.

Copper:zinc ratio – The treatment effect was not statistically significant. GLM with Repeated measures indicated that there were no significant differences between timepoints in either group A or B (Figure 5).

#### Essential Fatty Acid Analysis

Mean plasma GLA, LA, ARA, and DGLA concentrations for groups A and B are presented in Table 6. FADS1 and FADS2 activity indices were determined from the ARA:DGLA and GLA:LA ratios, respectively, and ELOVL5 activity index from the DGLA:LA ratio as previously described (14).

**Table 6:** Plasma essential fatty acid concentrations at all timepoints^¶^.

	**Timepoint 1**		**Timepoint 2**		**Timepoint 3**		**Timepoint 4**		**Timepoint 5**	
	**Mean**	**SD**	**Mean**	**SD**	**Mean**	**SD**	**Mean**	**SD**	**Mean**	**SD**
**Intervention group A**	**(*****N*** **=** **24)**		**(*****N*** **=** **22)**		**(*****N*** **=** **23)**		**(*****N*** **=** **21)**		**(*****N*** **=** **23)**	
GLA, mM	0.024	0.011	0.035	0.016	0.032	0.016	0.026	0.011	0.023	0.010
LA, mM	1.421	0.216	1.287	0.197	1.141	0.131	0.957	0.103	0.885	0.086
ARA, mM	0.676	0.195	0.705	0.203	0.679	0.120	0.529	0.100	0.485	0.107
DGLA, mM	0.164	0.056	0.166	0.055	0.167	0.050	0.134	0.041	0.119	0.030
**Intervention group B**	**(*****N*** **=** **22)**		**(*****N*** **=** **22)**		**(*****N*** **=** **18)**		**(*****N*** **=** **21)**		**(*****N*** **=** **22)**	
GLA, mM	0.026	0.009	0.028	0.009	0.041	0.019	0.027	0.012	0.022	0.009
LA, mM	1.457	0.283	1.301	0.196	1.119	0.170	0.978	0.219	0.858	0.112
ARA, mM	0.696	0.193	0.706	0.186	0.682	0.130	0.573	0.160	0.469	0.084
DGLA, mM	0.169	0.040	0.157	0.033	0.196	0.070	0.148	0.050	0.118	0.038

*Linoleic acid (LA); 18:3n−6 γ -linolenic acid (GLA); 20:3n−6 dihomo-γ -linolenic acid (DGLA); 20:4n−6 arachidonic acid (ARA)*.

¶ *Timepoint 1 = baseline; Timepoint 2 = week 4 of period 1; Timepoint 3 = week 8 of period 1; Timepoint 4 = week 4 of period 2; Timepoint 5 = week 8 of period 2*.

**Figure 6:**
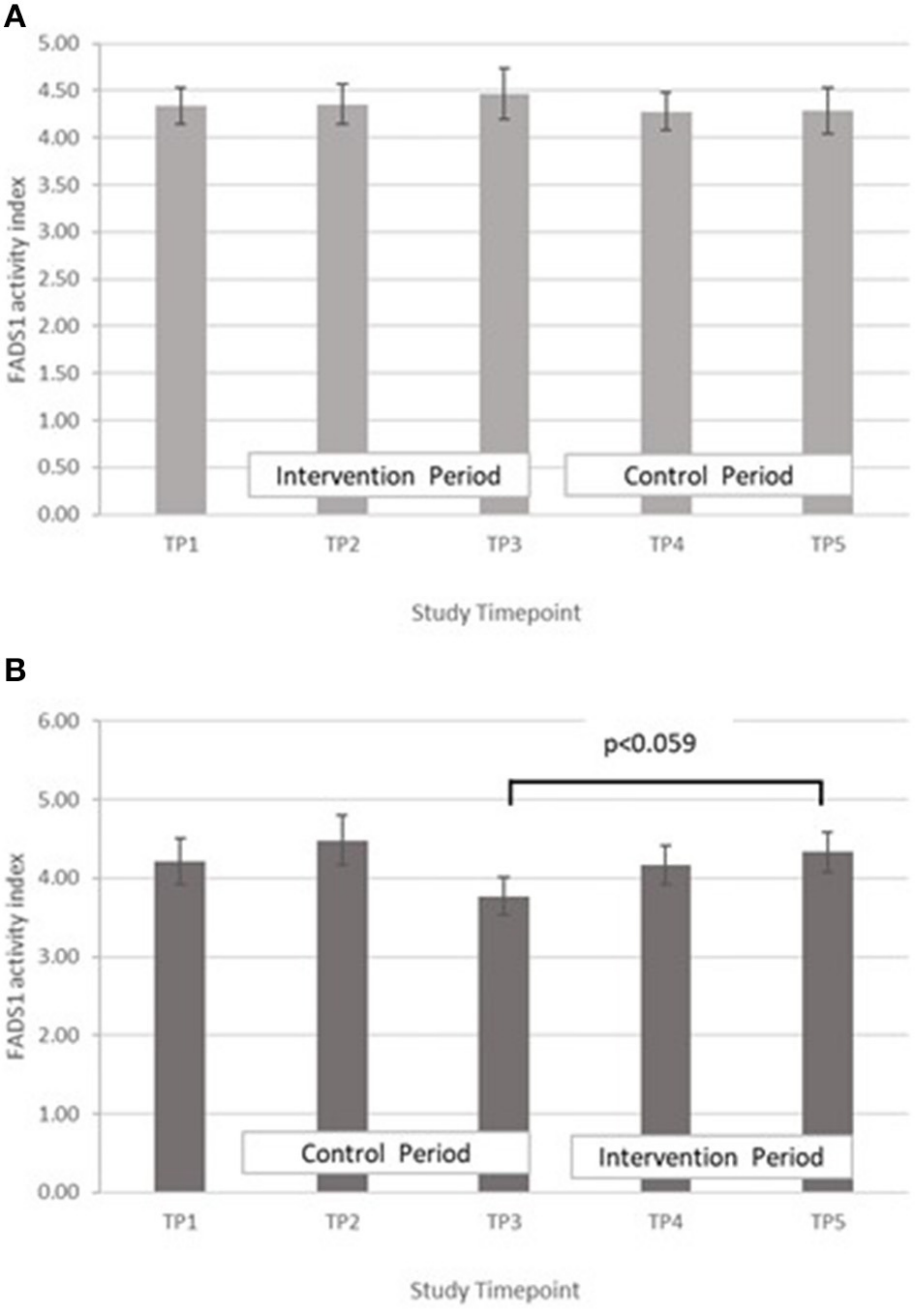
**(A)** Fatty acid desaturase 1 activity measured in Group A at baseline (T1), week 4 of period 1 (T2), week 8 of period 1 (T3), week 4 of period 2 (T4) and week 8 of period 2 (T5). Bars indicate estimated marginal means with standard error. **(B)** Fatty acid desaturase 1 activity measured in Group B at baseline (T1), week 4 of period 1 (T2), week 8 of period 1 (T3), week 4 of period 2 (T4) and week 8 of period 2 (T5). Bars indicate estimated marginal means with standard error.

FADS1: GLM with Repeated measures indicated that in group A there were no significant differences between timepoints T3 and T5, or T2 and T4. In Group B there was an increase in FADS1 activity index during the intervention period (between T 3 and 5) that came close to significance, *p* = 0.059, however this followed a marked dip in FADS1 activity at T3 (Figure 6). There were no significant differences in group B between T2 and T4. Pearson's correlation analyses showed that there were no significant linear associations between PZC and FADS1 at T3 (*r* = 0.04, *p* = 0.82) and T5 (*r* = 0.02, *p* = 0.91).

**Figure 7:**
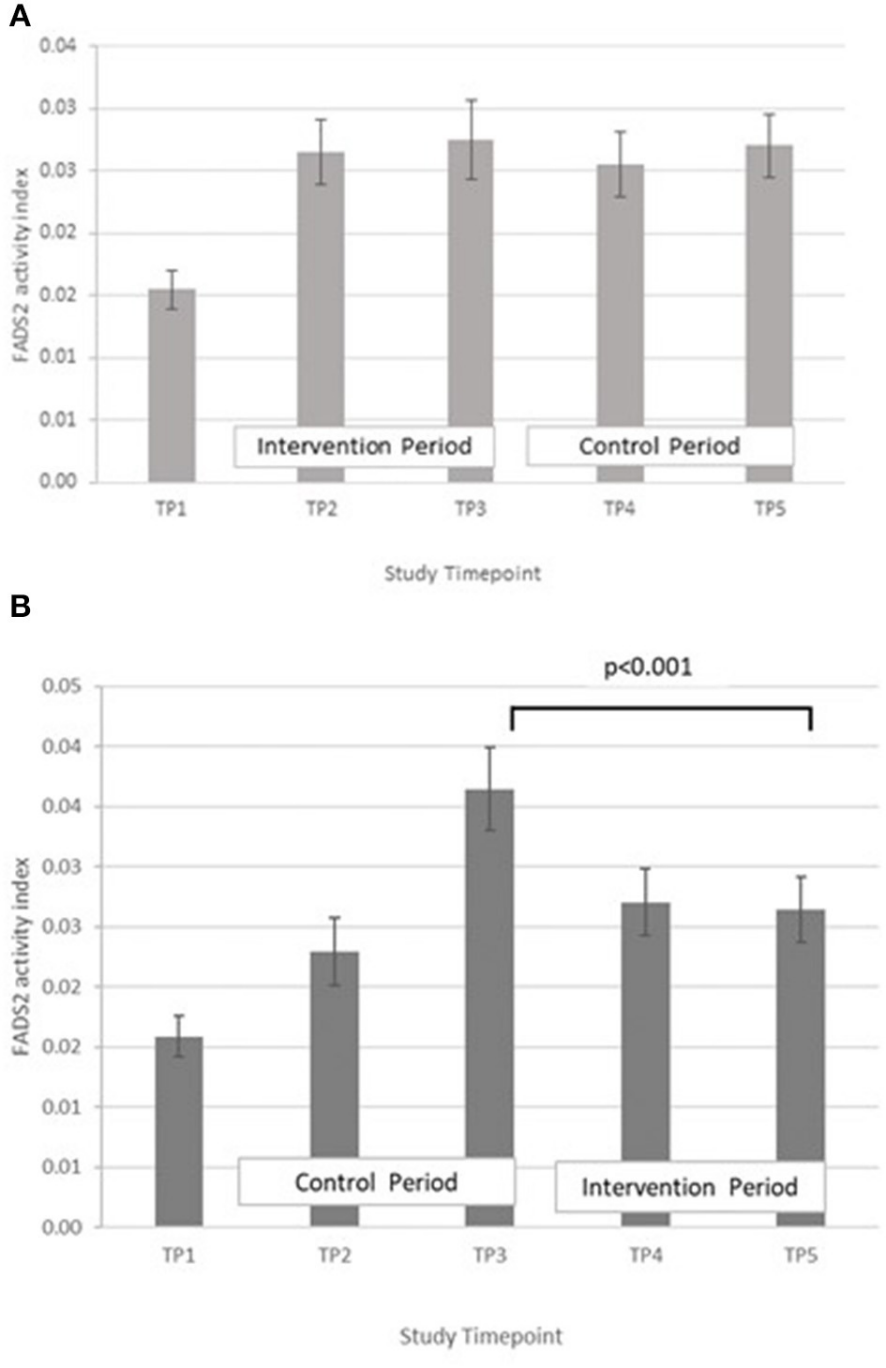
**(A)** Fatty acid desaturase 2 activity measured in Group A at baseline (T1), week 4 of period 1 (T2), week 8 of period 1 (T3), week 4 of period 2 (T4) and week 8 of period 2 (T5). Bars indicate estimated marginal means with standard error. **(B)** Fatty acid desaturase 2 and elongase-5 activity measured in Group B at baseline (T1), week 4 of period 1 (T2), week 8 of period 1 (T3), week 4 of period 2 (T4) and week 8 of period 2 (T5). Bars indicate estimated marginal means with standard error.

FADS2, GLA:LA ratio: GLM with Repeated measures indicated that there were no significant differences between timepoints in group A. In group B, FADS2 activity was significantly lower at T5 compared with T3 (*p* < 0.001) (Figure 7).

**Figure 8:**
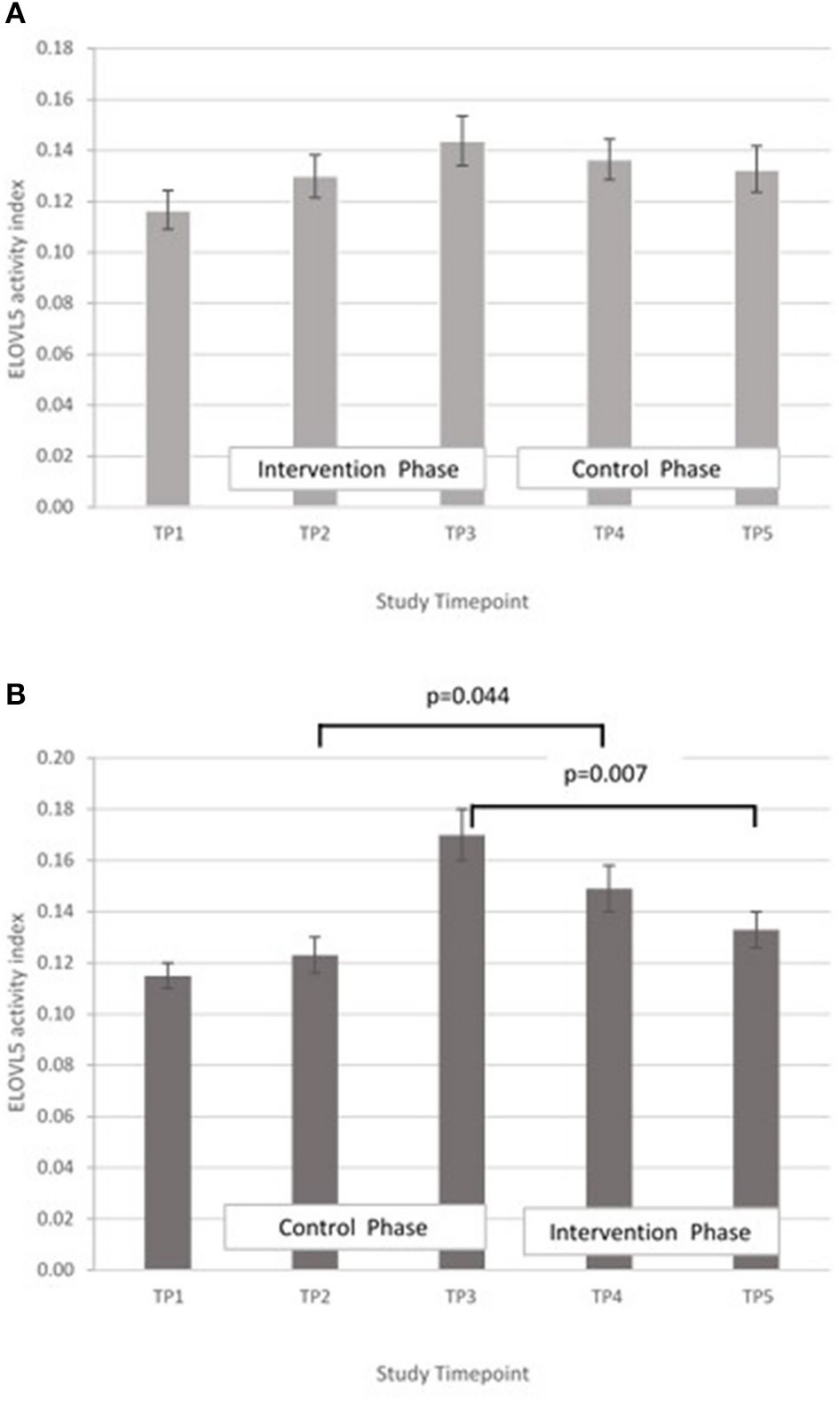
**(A)** Elongase-5 activity measured in Group A at baseline (T1), week 4 of period 1 (T2), week 8 of period 1 (T3), week 4 of period 2 (T4) and week 8 of period 2 (T5). Bars indicate estimated marginal means with standard error. **(B)** Elongase-5 activity measured in Group B at baseline (T1), week 4 of period 1 (T2), week 8 of period 1 (T3), week 4 of period 2 (T4) and week 8 of period 2 (T5). Bars indicate estimated marginal means with standard error.

ELOVL5, DGLA:LA ratio. GLM with repeated measures indicated no between timepoints in group A. In group B, ELOVL5 activity was significantly lower at T5 compared with T3 (*p* < 0.002) and higher in T4 compared with T2 (*p* = 0.012) (Figure 8).

#### Routine Haematology and Adverse Effects

No adverse physical adverse effects attributable to the consumption of either the control or intervention flours were reported during the study. Routine haematological indices are summarised in the Supplementary Table 1 and fell within normal ranges at all timepoints. Following the observation that plasma zinc, selenium and copper concentrations all fell significantly at T5, further investigations into the haematological measures were made, specifically between T4 and T5. GLM repeated measures revealed a significant fall in Hc from T4 to T5 in both groups (group A, *p* = 0.048; group B, *p* = 0.003). This was accompanied by a fall in Hb, but only statistically significant in group B (group A, *p* = 0.085; group B, *p* = 0.027). There were no significant changes in MCV, or MCHC. There was a significant fall in RBC in both groups from T4 to T3 (group A, *p* = 0.042; group B, *p* = 0.03).

## Discussion

Biofortification of a staple food is potentially a low-cost, sustainable mechanism of increasing the zinc intake of a population, and for Pakistan, wheat has been selected as the target staple as wheat flour is used for bread that is consumed with every meal. Zincol-2016 wheat was grown specifically for this study under optimal conditions of zinc fertiliser application and was able to achieve a mean zinc concentration of 49.3 mg/kg. The study showed that replacement of non-biofortified flour with flour milled from Zincol-2016 has the potential to increase dietary zinc intake by 6.0 mg per day, based on the quantities of flour typically consumed by the study participants, depending on the proportion of the bran removed prior to consumption. This is a marked increase in daily zinc intake, against a mean daily intake of 10 mg per day in the study participants (Table 1). This compares favourably with a study of agronomically biofortified wheat conducted in India, where addition of zinc rich fertiliser to the foliage resulted in a grain zinc concentration of 30 mg/kg, which translated into an increase of 3 mg per day in the diet of WRA (33). The authors stated that grains should contain between 40 and 60 mg Zn/kg in order to meet the RNI of 15 mg/d. The Zincol-2016 grain zinc content produced in the present study fell within this range, although there was some variability with zinc content ranging from 27.3 to 61.3 mg/kg. It is common practise with HHs in this community to sieve flour prior to use, depending on the type of bread that it is being used for. For Paratha, an oily bread eaten for breakfast, white flour is preferred, whereas for Naan and Roti, a mixed flour is often used. The fractional zinc absorption (FZA) from food depends on the bioavailability which is largely determined by the phytate content of the diet (34). The phytate content of the Zincol-2016 flour is comparable to that of standard varieties (MR Broadley, unpublished data) thus the total amount absorbed from Zincol-2016 is likely to be greater than that of standard varieties due to the higher total zinc content. Further studies on zinc bioavailability from foods made from Zincol-2016 flour are needed to confirm this. In terms of generalizability, the increase in dietary zinc intake calculated from the grain zinc content is presented as a range, depending on the bran content of the flour consumed. Flour consumption for the WRA was estimated from local bread recipes and the amount of bread consumed per day recorded in the 24-h dietary recalls. The mean grain consumption arrived at from this method, 224 g/d, is lower than the estimated national per capita mean grain consumption of 124 kg per year (340 g/d), thus the potential contribution of zinc biofortified grain to the daily zinc intake of the general population of Pakistan could be higher than our estimates for WRA.

To inform the scale of the release of biofortified varieties on a national level, it is important to be able to demonstrate the effect on key indicators of nutritional status and health outcomes. To that end, we examined outcomes arising from consuming biofortified flour for 8 weeks compared with a standard variety in a double-blind cross over RCT. Within group comparisons revealed that there was a significant increase in PZC at the midpoint of the intervention period (4 week) compared with the midpoint of the control (Figure 2). However, the difference was not present at the end of the intervention period. A similar finding was reported by Aaron et al. who conducted a study to investigate the impact of consuming of fortified wheat bread on PZC in healthy Senegalese men (35). The RCT had 4 treatment arms: a moderate and a high zinc fortification arms, in which participants were provided with fortified bread containing 7.5 mg zinc or 15 mg zinc, respectively, for 4 weeks which were compared with a control arm (bread without added zinc) and a liquid zinc supplement (15 mg zinc). Fasting blood samples taken at the mid and endpoint of the intervention, and the authors reported that across all timepoints the zinc supplemented group was the only group where PZC increased from baseline. In addition, a RCT of zinc biofortified wheat in India where WRA consumed zinc biofortified flour 6 months also failed to demonstrate an increase in PZC compared to the control, despite a significant improvement in self-reported morbidity (33). Zinc present in blood plasma exchanges rapidly with the liver and other tissues where zinc is required for various functions including metabolism, immune response and protein synthesis (36). In chronic zinc deficiency, it is plausible that following a modest increase in dietary zinc, the additional absorbed zinc is rapidly distributed to restore metabolic functions while PZC remains low, at least until deficiency is more fully resolved. Pinna et al. explored the response of PZC and the exchangeable zinc pool (EZP) of which plasma zinc is a component, to dietary zinc depletion and repletion (37). Participants were provided with a diet containing 13.7 mg zinc per day during the 5-week baseline and repletion phases. Following the baseline period, a moderately deficient diet containing 4.6 mg per day was provided for a 10-week depletion period. The findings indicated that neither PZC nor the size of the EZP responded to this modest (9.1 mg/d) reduction or increase in dietary zinc intake over this time period. Further studies of the longer-term effect of modest increases in dietary zinc intake on PZC following chronic deficiency are needed to explore the homeostatic response and changes in the size of the EZP.

In both groups, a significant decrease in PZC was observed in both groups at T5 (Figure 2). This decrease was also observed in the other minerals measured (Table 4; Figures 3–5), suggesting either a haemodilution effect or a systematic analytical error. Sample batches were randomised for timepoint, therefore a systematic analytical error at T5 is unlikely. In addition, the EFA concentrations also follow a similar trend (Table 5), with the lowest values at T5 in both groups. For EFA analysis, sample batches were also randomised for timepoint, and samples were analysed in duplicate and the duplicates were randomised. Further investigation of haematological indices haematocrit (Hc) and haemoglobin concentration, indices of hydration status, also revealed a significant fall in Hc, Hb concentration and RBC between T4 and T5, all of which are consistent with haemodilution. The study took place from October to February 2019 with January and February being the coolest month of the year in Northern Pakistan. Data for T4 and T5 were collected in mid-January and mid-February, respectively, meaning that an impact of a difference in ambient temperature between these 2 timepoints on hydration status is unlikely. There is no evidence from the 24 h recalls for an increase in fluid intake across the 5 Ts. The reason for this fall in blood parameters at T5 therefore remains unclear. Plasma Cu:Zn ratios have also been suggested as an indicator of zinc status (38, 39), with an optimal Cu:Zn ratio of 0.7–1.00 and ratios above 1.5 reflecting an inflammatory response or a decreased Zn status (40). The mean Cu:Zn ratios for the study participants were above or close to 1.5 at all Ts in both groups, and commensurate decreased zinc status as suggested by the low dietary zinc intakes, and low plasma zinc concentrations.

The proxy indices for FADS1, FADS2, and ELOVL5 activities were estimated from the ratio of product to precursor in the pathway involving desaturation and elongation of linoleic acid (18:2 n-6) to form arachidonic acid (20:4 n-6) (14). Massih et al. examined the effect of zinc supplementation (25 mg per day for 13 days) with and without food on FADS1, FADS2, and ELOVL5 activity indices in adult men (14). After adjusting for baseline, they reported a significantly higher FADS1 activity index in participants consuming zinc with a meal, than in those consuming zinc without food. They did not report any significant difference in FADS2 activity index between the two groups and suggested that FADS2 may be less sensitive to changes in zinc nutriture than FADS1. The ELOVL5 activity index tended to increase when zinc was consumed without a meal, but did not reach significance. Data from the present study suggest a rise in FADS1 activity with increased zinc intake, however it failed to reach statistical significance (Figure 6). Some significant changes in FADS2 and ELOVL5 activity were observed in group B only but were inconsistent across the study periods (Figures 7, 8) In a study of healthy human volunteers, the LA:DGLA ratio was reported to be significantly higher in participants with relative low dietary intakes when compared with those with higher dietary zinc intakes (41). Similarly, an *in vivo* study using a chicken model (Gallus Gallus) reported that the LA:DGLA ratio measured in erythrocytes was higher in chickens fed a low zinc diet, compared to those consuming a zinc biofortified diet (42). This suggests a decrease in the activity of either FADS2 or ELOVL5 or both enzymes when dietary zinc is low. This would be consistent with an increase in FADS2 and/or EVOL5 when zinc biofortified flour was consumed in the present study, which was seen in group B at the mid- point of the biofortified flour period (T4) compared with the midpoint of the control flour period (T2), however the reverse was seen at the end of the biofortified period (T5), compared with the end of the control flour period (T3). The blood samples taken for all biochemical analyses were non-fasting, in contrast to previous studies that have reported a relationship between fatty acid metabolism and zinc nutriture from samples taken during the fasted state (14, 17). Perturbations in lipid profiles in response to a recent meal may be masking any subtle changes in EFA ratios due to FADS or ELOVL5 activity in the present study.

A strength of the study is that, to our knowledge, this is the first study that explores the efficacy of a zinc biofortified strain of wheat, Zincol-2016, to improve zinc intake and status in Pakistan. It also provides valuable data on the proposed novel indicators of zinc status, FADS1, FADS2, and ELOVL5, following modest increases in dietary zinc intake in a low resource community setting. The study was double-blind and the cross over design enabled repeated measures analysis under both the intervention and control arms of the study in all participants which enhanced the statistical power in comparison to a two-arm study without cross over as each subject acted as her own control. This provided mitigation to some extent of the limitation of a small sample size, as demonstrated by the power calculation. Another limitation was the relatively short duration of the intervention (8 weeks). In addition, we had intended to measure inflammatory markers of zinc status (CRP and AGP) so that PZC adjustments for the presence of infection could be undertaken, however this was not possible for technical reasons. Ideally, blood samples should be collected in the fasted state to improve consistency and reproducibility of blood biochemical parameters. This was not possible in the present study for logistical and cultural reasons. The study was not powered a priori to detect changes in FADS or ELOVL5 activities, therefore results should be interpreted within this exploratory context. Finally, the high-zinc variety of wheat evaluated in this study, Zincol-2016, was grown under optimal conditions of fertiliser application on a single farm in Punjab province. An effectiveness trial is currently underway to assess the potential of Zincol-2016 to increase dietary zinc intake when grown by farmers living in the vicinity of the study population in KP Province, with some technical support for zinc fertiliser application. Further work is underway to examine Zincol-2016 grain zinc content when grown in different soil conditions, with and without the use of zinc fertiliser.

In summary, the results of this study demonstrate that consuming zinc biofortified flour can have a marked impact on total dietary zinc intake in rural, communities, where diet diversity is low, and there is a reliance on a limited number of plant-based foods and staples to meet energy needs. An increase in the daily zinc intake of between 3.0 and 6.0 mg, did not lead to a sustained, measurable increase in PZC. Some interesting trends towards an increase in FADS activity with increased zinc intake were observed but these did not achieve statistical significance. ELOVL5 activity changed significantly, but inconsistently across the study timepoints. Further studies with a larger sample size and intervention duration are needed to further investigate whether FADS1 and FADS2 activities, estimated from plasma fatty acid ratios, respond sensitively and reliably to modest changes in daily zinc intake.

## Data Availability Statement

The raw data supporting the conclusions of this article will be made available by the authors, without undue reservation.

## Ethics Statement

The studies involving human participants were reviewed and approved by STEMH Ethics Committee, University of Central Lancashire, and Khyber Medical University Ethics Committee. The patients/participants provided their written informed consent to participate in this study.

## Author Contributions

NL, MK, and MZ initiated and conceptualised this RCT within the parent BiZiFED program. MB, HM, EJ, and MHZ were involved in the overall program design including agronomic management of biofortified and reference wheat crop management. JSi and ST performed data analysis. GK collected the dietary data. AB performed the dietary analysis. BS and UU managed the handling of the blood samples and field data collection. HO managed the database and provided support and liaison with the field management team. EB and SY performed the mineral and grain analyses. JK and JSu performed the EFA analyses and were involved in the data interpretation. NL drafted the manuscript. All authors were involved in the revision of the manuscript.

## Funding

This BiZiFED project was funded by Biotechnology and Biological Sciences Research Council (BBSRC) Global Challenges Research Fund, Foundation Awards for Global Agriculture and Food Systems Research, Grant Number BB/P02338X/1. The Zincol-2016 seed was supplied by HarvestPlus and grown by Fauji Fertilizer Company. The Abaseen Foundation Pakistan facilitated the use of the health centre and access to the community. Funding for the fatty acid analyses was provided by HarvestPlus. The funders were not involved in the design, conduct, or analysis of the trial.

## Conflict of Interest

MHZ is employed by Fauji Fertilizer Company. The remaining authors declare that the research was conducted in the absence of any commercial or financial relationships that could be construed as a potential conflict of interest.

## Publisher's Note

All claims expressed in this article are solely those of the authors and do not necessarily represent those of their affiliated organizations, or those of the publisher, the editors and the reviewers. Any product that may be evaluated in this article, or claim that may be made by its manufacturer, is not guaranteed or endorsed by the publisher.
