# Supplementary material for: Biofortified Wheat Increases Dietary Zinc Intake: A Randomised Controlled Efficacy Study of Zincol-2016 in Rural Pakistan
Source: Front Nutr. 2022 Jan 18;8:809783. doi: 10.3389/fnut.2021.809783 (PMC8804315; doi:10.3389/fnut.2021.809783)
Supplement: Supplementary file 1 [file Table_1.DOCX]

Supplementary Table 1. Haematological Indices at all timepoints^¶^

|  | *Timepoint 1* | | *Timepoint 2* | | *Timepoint 3* | | *Timepoint 4* | | *Timepoint 5* | |  |
| --- | --- | --- | --- | --- | --- | --- | --- | --- | --- | --- | --- |
|  |  |  |  |  |  |  |  |  |  |  |  |
|  | Mean | SD | Mean | SD | Mean | SD | Mean | SD | Mean | SD |  |
| Intervention group A | *(N=24)* | | *(N=25)* | | *(N=22)* | | *(N=23)* | | *(N=23)* | |  |
| Haematocrit (%) | 39.9 | 5.4 | 37.7 | 3.7 | 38.1 | 2.9 | 38.4 | 3.7 | 37.2 | 4.1 |  |
| Haemoglobin (g/dL) | 13.1 | 2.2 | 12.8 | 1.5 | 12.9 | 1.2 | 12.9 | 1.6 | 12.5 | 1.7 |  |
| Mean corpuscular volume (fL) | 82.6 | 6.2 | 80.8 | 6.1 | 82.1 | 5.5 | 82.3 | 6.4 | 82.0 | 6.6 |  |
| MCHC (g/dL) | 32.8 | 2.0 | 33.7 | 1.9 | 33.4 | 2.8 | 33.0 | 2.8 | 33.6 | 1.9 |  |
| Red blood cell count (million /mm^3^) | 4.8 | 0.7 | 4.7 | 0.6 | 4.7 | 0.6 | 4.8 | 0.6 | 4.7 | 0.7 |  |
|  |  | |  | |  | |  | |  | |  |
| Intervention group B | *(N=24)* | | *(N=22)* | | *(N=23)* | | *(N=22)* | | *(N=20)* | |  |
| Haematocrit (%) | 39.0 | 3.1 | 38.2 | 2.8 | 39.2 | 2.9 | 39.5 | 3.2 | 37.9 | 3.7 |  |
| Haemoglobin (g/dL) | 12.7 | 1.0 | 12.9 | 1.1 | 13.2 | 0.9 | 13.1 | 1.1 | 12.6 | 1.1 |  |
| Mean corpuscular volume (fL) | 83.3 | 8.1 | 81.8 | 8.2 | 82.9 | 8.2 | 83.1 | 8.6 | 83.8 | 9.0 |  |
| MCHC (g/dL) | 32.3 | 1.5 | 33.7 | 1.5 | 33.6 | 2.5 | 33.9 | 2.3 | 33.6 | 1.5 |  |
| Red blood cell count (million /mm^3^) | 4.7 | 0.5 | 4.7 | 0.6 | 4.7 | 0.5 | 4.7 | 0.5 | 4.5 | 0.6 |  |
|  |  |  |  |  |  |  |  |  |  |  |  |

^¶^Timepoint 1 = baseline; Timepoint 2 = week 4 of period 1; Timepoint 3= week 8 of period 1; Timepoint 4= week 4 of period 2; Timepoint 5= week 8 of period 2
